# In vivo CHI3L1 (YKL-40) expression in astrocytes in acute and chronic neurological diseases

**DOI:** 10.1186/1742-2094-7-34

**Published:** 2010-06-11

**Authors:** Dafna Bonneh-Barkay, Guoji Wang, Adam Starkey, Ronald L Hamilton, Clayton A Wiley

**Affiliations:** 1 Department of Pathology, University of Pittsburgh, 200 Lothrop St. Pittsburgh, PA 15213, USA

## Abstract

**Background:**

CHI3L1 (YKL-40) is up-regulated in a variety of inflammatory conditions and cancers. We have previously reported elevated CHI3L1 concentration in the cerebrospinal fluid (CSF) of human and non-human primates with lentiviral encephalitis and using immunohistochemistry showed that CHI3L1 was associated with astrocytes.

**Methods:**

In the current study CHI3L1 transcription and expression were evaluated in a variety of acute and chronic human neurological diseases.

**Results:**

ELISA revealed significant elevation of CHI3L1 in the CSF of multiple sclerosis (MS) patients as well as mild elevation with aging. *In situ *hybridization (ISH) showed CHI3L1 transcription mostly associated with reactive astrocytes, that was more pronounced in inflammatory conditions like lentiviral encephalitis and MS. Comparison of CHI3L1 expression in different stages of brain infarction showed that YKL40 was abundantly expressed in astrocytes during acute phases and diminished to low levels in chronic infarcts.

**Conclusions:**

Taken together, these findings demonstrate that CHI3L1 is induced in astrocytes in a variety of neurological diseases but that it is most abundantly associated with astrocytes in regions of inflammatory cells.

## Background

CHI3L1 (chitinase 3-like protein 1, human cartilage glycoprotein 39 (HC-gp39), YKL-40, gp38k, chondrex, breast regression protein 39 (BRP-39)) is up-regulated in inflamed tissues in ulcerative colitis, Crohn's disease, rheumatoid arthritis, osteoarthritis, asthma, chronic obstructive pulmonary disease (COPD) and liver cirrhosis, as well as in solid cancers [1-10]. Recently, we have shown that CHI3L1 expression is induced in the CNS of human and non-human primates with lentiviral encephalitis (HIV encephalitis (HIVE) and SIVE) [11]. The physiological role of CHI3L1 is not known, however, it has been hypothesized to be involved with tissue remodeling during inflammation. In a recent study, BRP-39 (the mouse homologue of human CHI3L1) knockout mice showed a blunted immune response to allergic sensitization (e.g. decreased accumulation of dendritic cells in the lungs, decreased IgE production, increased percentage of apoptotic T-cells and macrophages in the bronchoalveolar lavage) accompanied by reduced peribronchial fibrosis and collagen content [12].

Previous studies have reported altered expression of CHI3L1 in CNS disorders. Brain tissue homogenate studies have found increased CHI3L1 mRNA in schizophrenia, autism and Alzheimer's disease [13-15]. CSF studies have also found elevated CHI3L1 in patients with purulent meningitis and encephalitis [16]. In our previous study, longitudinal analysis of CSF of SIV-infected macaques showed that CHI3L1 concentrations in the CSF increased between 2 to 8 weeks before terminal encephalitis. Additionally, CSF levels were found to be 10-fold higher than plasma levels. Immunohistochemical analysis showed that CHI3L1 was associated with astrocytes around microglial nodules in SIVE and occasional activated macrophages/microglia. We have shown that CHI3L1 can inhibit bFGF signaling through FGFR1 and inhibit bFGF-induced axonal branching in hippocampal neurons [11]. Thus, CHI3L1 potentially has the capacity to modulate neurotrophic factor associated changes in the functionality, plasticity or regenerative ability of neurons.

The current study was carried out to identify the spectrum of CHI3L1 expression in a variety of neurodegenerative and neurological diseases, the cellular origin of CNS CHI3L1, and in the case of brain infarction a comparison between acute and chronic expression. We show that CHI3L1 is elevated in CSF from patients with MS and to a lesser extent with aging. CHI3L1 transcription is induced in reactive astrocytes and is associated with local neuroinflammation in acute and chronic diseases.

## Methods

### Human specimens

Ten de-identified CSF samples from each of AD, MS, ALS, stroke and normal control patients were obtained from the Human Brain and Spinal Fluid Resource Center (UCLA, CA) and the Center for ALS Research at the University of Pittsburgh. Cortical tissue samples from three cases of AD and schizophrenia, two cases of infarct and Pick's disease and one case of MS and ALS were obtained from the Human Brain and Spinal Fluid Resource Center (UCLA, CA) and used for ISH and immunohistochemical analyses. All human studies were approved by the corresponding institutional review boards.

### Pigtailed macaque tissues

Previously banked brains from four pigtailed macaques (*Macaca nemestrina*) infected with SIVDeltaB670 viral swarm (SIVdB670) that developed encephalitis and three macaques that did not develop encephalitis were used for *in situ *hybridization (ISH) and immunohistochemistry.

### In situ hybridization

Sense and antisense CHI3L1 DNA templates containing the T7 promoter were generated by PCR from the pUC57 vector (GenScript, Piscataway, NJ) containing the full length YKL40 cDNA (NCBI Reference Sequence: NM_001276.2). ^35^S-labeled RNA probes were generated using MAXIscript in vitro transcription kit (Ambion, Austin, TX). After removal of paraffin, cortical tissue sections from patients with MS, AD, Pick's disease, ALS, stroke and schizophrenia and SIV-infected pigtailed macaques were processed for *in situ *hybridization (ISH) and then for immunohistochemistry. For ISH, tissue sections were microwave oven treated and then incubated in hybridization buffer (1× HYB buffer, 0.6 M NaCl, 10% dextran, 50 μg/ml tRNA, 0.1 M DTT) containing radiolabeled CHI3L1 probe (50,000 cpm/μl) in 50°C over night. The following day the tissue sections were washed and processed for immunofluorescence. Tissue sections were exposed to emulsion (Eastman Kodak company, Rochester, NY) for 10 days in 4°C and then ISH signal was visualized using D19 (Sigma-Aldrich, St. Louis, MO) and then fixed in Rapid fix (Sigma-Aldrich, St. Louis, MO).

### Immunofluorescence

Formalin-fixed, paraffin-embedded, 6-micrometer thick tissue sections were deparaffinized in Histoclear (National Diagnostics, Atlanta, GA) and rehydrated for 3 minutes in 100%, 95%, and 70% alcohol followed by PBS. Endogenous peroxidase activity was inactivated by immersing the section in 3% H_2_O_2 _for 20 minutes. Antigen unmasking was performed using antigen retrieval Citra^® ^solution (BioGenex, San Ramon, CA). Tissue sections were blocked with protein blocking agent (Thermo, Pittsburgh, PA) for 20 minutes. GFAP staining was performed using polyclonal rabbit anti-human GFAP (1:500; DAKO, Carpinteria, CA). Iba1 staining was performed using rabbit anti-human Iba1 (1:500; Waco Chemicals, Richmond, VA) and CHI3L1 staining was performed using goat anti-human chitinase 3-like 1 (1:1000; R&D systems, Minneapolis, MN). These staining were followed by goat anti-rabbit Dylight 488 antibody (1:200; Jackson ImmunoResearch Laboratories, West Grove, PA) or mouse anti-goat biotin-conjugated antibody (1:200; Jackson ImmunoResearch Laboratories, West Grove, PA).

### Quantitation of in situ hybridization and immunofluorescence

Five fields from each case were captured by confocal microscopy and analyzed for the number of ISH-positive cells or ISH/IHC pixels per field (LSM 510, Carl Zeiss MicroImaging, Inc., Thornwood, NY). Illumination was provided by Argon (458, 477, 488, 514 nm, 30 mW), HeNe (543 nm, 1 mW) lasers. Digital images were captured with LSM 510 version 4.2 (Zeiss).

### Semi-quantitation of CHI3L1 in situ hybridization in infarct cases

Infarct presence or absence was established by a neuropathologist (RLH), then infarct age was charachterized as acute, subacute, or chronic based upon neuropathological features seen on hematoxylin staining as previously described [17]. Due to the focal nature of infarct pathology a semi-quantitative scoring was performed by a neuropathologist (CAW) blinded to clinical characteristics in 4 cases of acute infarct, 5 cases of subacute infarcts and 5 cases of chronic infarct. ISH scoring of CHI3L1 was assigned a score from 0 to (++++) based on ISH intensity: no siganl = 0, occasional positive cells = (+/-), weak signal = (+), moderate signal = (++), strong signal = (+++) and intense signal = (++++).

### CHI3L1 enzyme-linked immunosorbent assay (ELISA)

CHI3L1 levels were determined, in duplicate, for all of the CSF samples using the MicroVue CHI3L1 ELISA kit from Quidel Corporation (San Diego, CA) according to the manufacturer's protocol. Absorbance was measured using a microplate reader (BioTek Instruments, Inc., Winooski, VT).

### Statistical analysis

Average ± standard error (SEM) of CSF CHI3L1 concentrations from MS, AD, ALS, stroke patients and healthy young and aged controls were graphed. The Mann-Whitney test was used to computes the statistical difference between disease groups and control patients. CHI3L1 ISH and GFAP imuunohistochemistry pixels were captured by confocal microscopy from 5 random fields of each of the SIVE, SIV, infarct, MS, AD, Pick's disease and schizophrenia cases. Average pixels per filed were calculated and a linear regression was used to determine a correlation between CHI3L1 pixel count and GFAP pixel count.

## Results

### CSF YKL40 elevation with aging and acute and chronic neurological diseases

**Figure 1:**
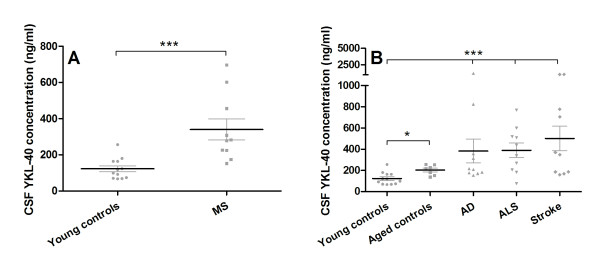
**CSF CHI3L1 levels in neurodegenerative diseases and stroke**. CSF samples from MS, AD, ALS, stroke and control patients were analyzed for CHI3L1 using the CHI3L1 ELISA kit. The Mann-Whitney test was used to evaluate group differences (*P < 0.05, **P < 0.001, ***P < 0.0001). CSF YKL-40 levels in MS patients were significantly elevated compared to young, age-matched controls (A). While all neurodegenerative diseases and stroke show difference from young controls, AD, ALS and stroke patients do not show significant differences when compared to older age-matched controls (B).

We have previously observed elevated CHI3L1 concentration in the CSF of SIV and HIV encephalitis [11] and therefore wanted to assess its concentration in a broad spectrum of human neurological diseases. CSF CHI3L1 levels of MS patients (age range: 25 to 58) were compared to young healthy controls (age range: <40) and were found to be significantly elevated (*p *= 0.0007) (Figure 1A, Table 1). CSF samples from healthy aged controls, AD, ALS and stroke showed statistically significant elevated levels of CHI3L1 compared to young controls. However there was no statistical difference between AD (age range: 59 to 86; median age: 75), ALS (age range: 32 to 74; median age: 62.5) and stroke (age range: 60 to 89; median age: 72) when these values were compared to healthy older age-matched controls (age range: 60 to 70, median age: 65). CSF CHI3L1 levels in healthy older people are significantly higher then healthy young controls (*p *= 0.0126) which implies that there is a modest but significant increase of CSF CHI3L1 with aging (Figure 1B, Table 1).

**Table 1:** CSF CHI3L1 levels in neurodegenerative diseases.

Condition (n)	Age range (median)	CHI3L1 concentration (range, ng/ml)	CHI3L1 concentration (median, ng/ml)
Healthy young controls (n = 12)	<40	67-256	109
MS (n = 10)	28-58 (48)	152-697	281
Aged healthy controls (n = 7)	60-70 (65)	139-257	218
AD (n = 10)	59-86 (75)	153-1230	212
ALS (n = 10)	32-74 (62.5)	75-769	388
Stroke (n = 10)	60-89 (72)	160-1065	359

CSF samples from MS, AD, ALS, stroke and control patients were analyzed for CHI3L1 using the MicroVue CHI3L1 ELISA kit.

### CHI3L1 transcript is found predominantly in astrocytes in neurological diseases

**Figure 2:**
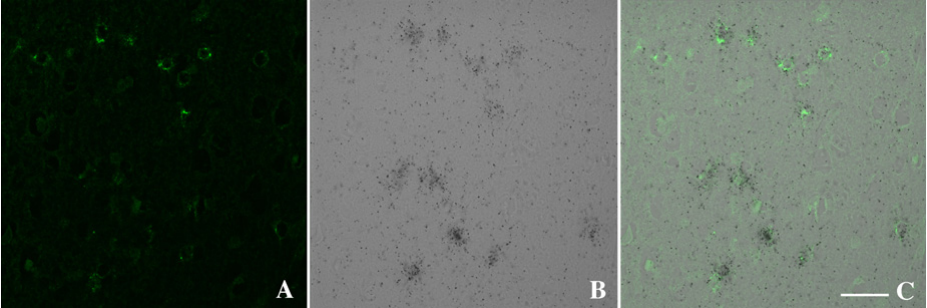
**CHI3L1 mRNA co-localizes with CHI3L1 immunohistochemistry in SIVE**. Six-μm-thick paraffin-embedded sections were hybridized with ^35^S-labeled RNA probe for CHI3L1 (B) followed by immunohistochemistry with for CHI3L1 (A) as described in Methods. CHI3L1 mRNA co-localizes with CHI3L1 immunohistochemistry, scale bar = 50 μm (C).

**Figure 3:**
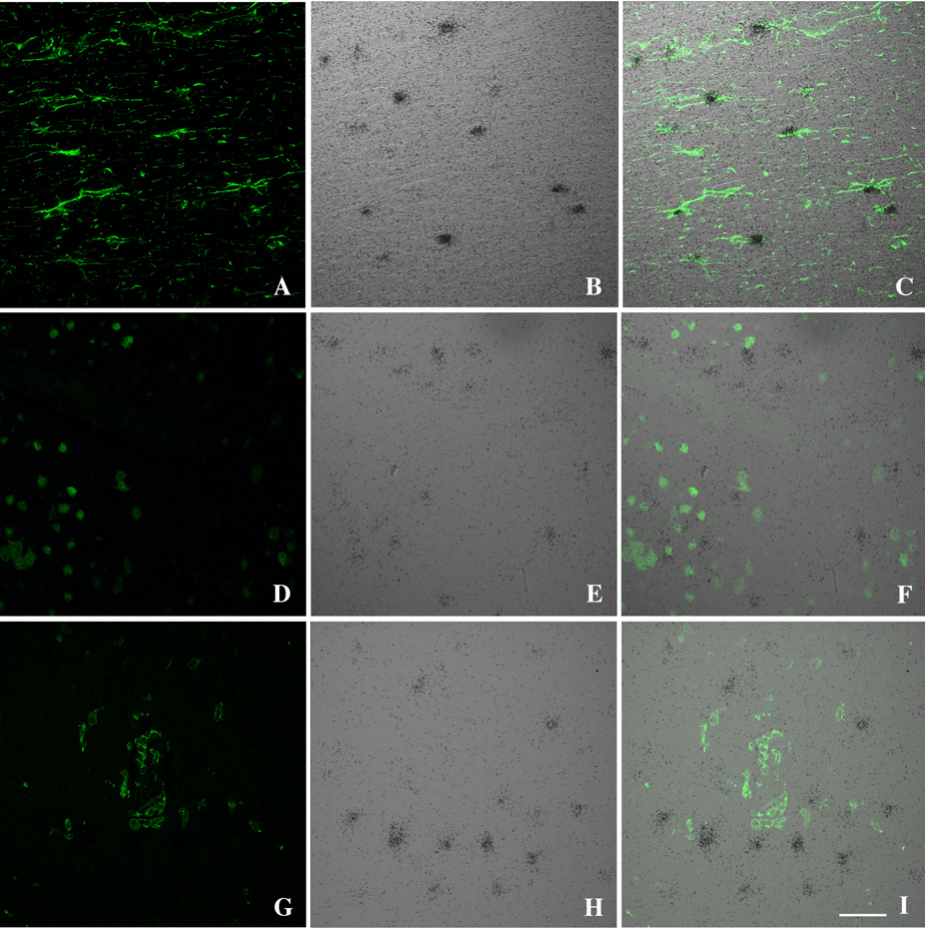
**YKL-4 mRNA co-localizes with GFAP staining in SIVE**. Six-μm-thick paraffin-embedded sections were hybridized with ^35^S-labeled RNA probe for CHI3L1 (middle panels) followed by immunohistochemistry with GFAP (A-C), NeuN (D-F) or Iba1 (G-I) as described in Methods. The right panels show the combined images of the ISH and immunohistochemistry, scale bar = 50 μm.

Since our previously published *in vitro *studies demonstrated that YKL40 was present in the supernatant of macrophages and not astrocyte or neuronal cultures, we were surprised to observe YKL40 staining localized with astrocytes in regions of microglial nodules and occasional activated macrophages/microglia in SIVE and HIVE. To elucidate the cellular source of CHI3L1 in SIVE encephalitis we performed ISH combined with immunohistochemistry. In order to validate the presence of CHI3L1 protein with its transcript we performed combined CHI3L1 ISH with immunohistochemistry for CHI3L1 on brain tissue from pigtailed macaques that developed SIVE. This confirmed that the antibody against CHI3L1 stained the cells that expressed the CHI3L1 transcript (Figure 2). The sense probe showed no significant hybridization (data not shown). Since the signal was more robust with ISH and we wanted to identify the cellular source of CHI3L1, we performed combined CHI3L1 ISH with immunohistochemistry for GFAP as a marker for astrocytes, NeuN as a marker of neurons and Iba1 as a marker for microglia/macrophages. CHI3L1 transcripts were shown to co-localize with GFAP staining (Figure 3A-C) but not with NeuN (Figure 3D-F) or Iba1 staining (Figure 3G-I) in SIVE.

**Figure 4:**
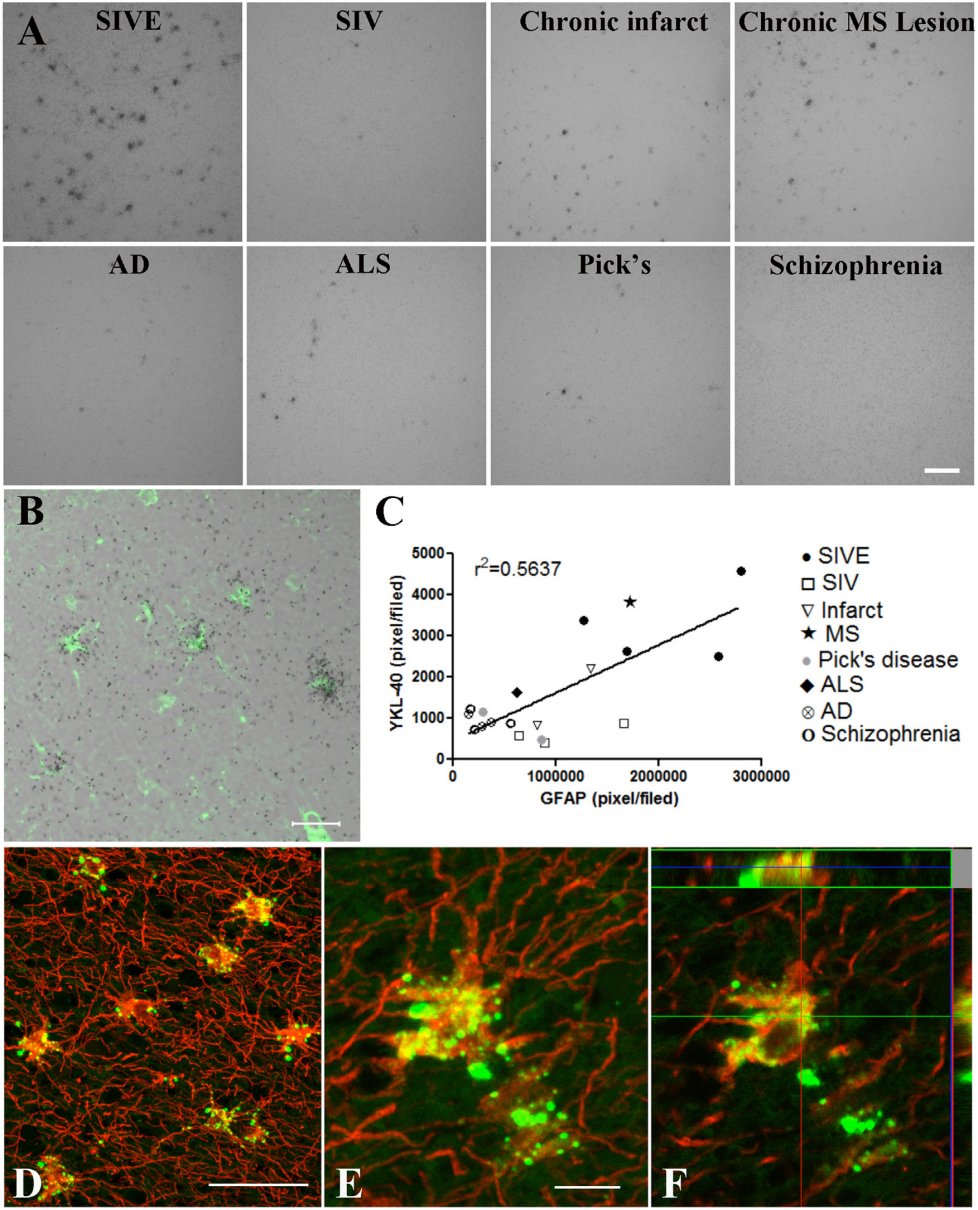
**CHI3L1 mRNA in neurological diseases**. Six-micrometer-thick paraffin-embedded sections from SIV encephalitis, SIV without encephalitis, AD, ALS, MS, Pick's disease, infarct and schizophrenia cases were hybridized with ^35^S-labeled RNA probe for CHI3L1 as described in Methods, scale bar = 100 μm (A). CHI3L1 ISH co-localized with GFAP in AD, scale bar = 20 μm (B) and brain infarction, scale bar = 50 μm (D). Higher magnification show co-localization in a single cell, scale bar = 10 μm (E). The panels to the right and on the top depict reconstructions from a confocal z-stack in xz and yz direction to confirm that CHI3L1 (green) co-localizes with GFAP (red) staining as seen in yellow (F). Five random fields from each case were captured by confocal microscopy and analyzed for CHI3L1 and GFAP pixels per field (C). Linear regression showed a positive correlation between CHI3L1 pixel count and GFAP pixel count (r^2 ^= 0.5637; black circle SIVE, white circle SIV, black star MS, white triangle infarct, black diamond ALS, crossed square AD, gray circle Pick's disease, white circle Schizophrenia).

These results suggest that while macrophages can synthesize YKL40 *in vitro, in vivo *CHI3L1 transcription is most commonly associated with astrocytes. Since we found elevated CHI3L1 levels in the CSF of a variety of neurological and neurodegenerative diseases, we wanted to study whether CHI3L1 expression is prevalent in astrocytes in those conditions. Combined ISH with GFAP staining was performed on sections from 4 cases of SIVE, 3 cases of SIV without encephalitis, AD and schizophrenia, 2 cases of Pick's disease and infarct and one case of MS and ALS. The ISH signal showed robust CHI3L1 transcription in SIVE and MS, focal ISH signal in chronic infarct cases while AD, ALS, Pick's disease and schizophrenia showed a few scattered positive cells in some areas while other areas were negative (Figure 4A). CHI3L1 ISH signal and immunohistochemistry was co-localized with GFAP staining in all the tested diseases (e.g. AD (Figure 4B) and brain infarction (Figure 4D). Confocal z-stack confirms that CHI3L1 (green) co-localizes with GFAP (red) staining as seen in yellow (Figure 4E &4F). Pixel counts of CHI3L1 ISH signal correlated with pixel counts of GFAP staining (r^2 ^= 0.5637) indicative of a correlation between CHI3L1 transcription and reactive gliosis (Figure 4C).

In summary, CHI3L1 expression in MS, aging and other neurological diseases is mostly associated with astrocytes. There is a correlation between CHI3L1 transcription and GFAP staining which is indicative of reactive gliosis. CHI3L1 transcription was more pronounced in diseases with a more pronounced inflammatory nature like encephalitis and MS.

#### YKL40 in acute and chronic stroke

**Figure 5:**
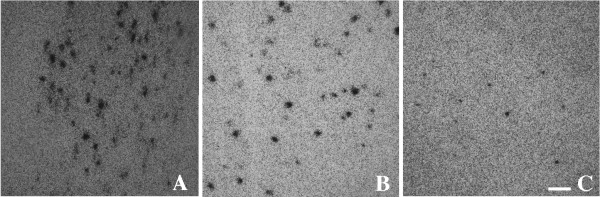
**CHI3L1 transcription in acute, subacute and chronic infarcts**. Six-micrometer-thick paraffin-embedded sections from acute (A), subacute (B) and chronic infarct (C) were hybridized with ^35^S-labeled RNA probe for CHI3L1 as described in Methods. Representative fields from acute, subacute and chronic infarcts were captured by confocal microscopy, scale bar = 50 μm.

The third aim of this study was to follow CHI3L1 expression in different stages of brain infarct. For that purpose we analyzed CHI3L1 transcription in acute, subacute and chronic brain infarction in humans. Combined ISH and GFAP staining showed CHI3L1 expression in astrocytes in the penumbra of the infarct that was more intense during the acute phase of infarction. Subacute cases showed less signal and chronic infarcts showed weak or no signal above background (Table 2, Figure 5).

**Table 2:** CHI3L1 ISH in acute, subacute and chronic infarcts.

Condition	**Case No**.	Age and sex	CHI3L1 ISH signal intensity
Acute infarct	1	58 female	+++
	2	61 male	++++
	3	79 female	++++
	4	85 male	++

Subacute infarct	1	56 female	+++
	2	81 female	0
	3	70 female	+
	4	89 female	+/-
	5	89 male	+++

Chronic infarct	1	88 female	+
	2	74 male	+
	3	45 male	++
	4	77 female	0
	5	88 female	0

Six-μm-thick paraffin-embedded sections were hybridized with ^35^S-labeled RNA probe for CHI3L1 as described in Methods. ISH signal intensity was scored in 4 cases of acute infarct, 5 cases of subacute infarcts and 5 cases of chronic infarct (intense signal (++++), strong signal (+++), moderate signal (++), weak signal (+), occasional positive cells (+/-) and no signal (0)).

## Discussion

In this study we found that CHI3L1 is expressed in astrocytes and is associated with reactive gliosis in different neuropathological conditions particularly those associated with neuroinflammation. Therefore CHI3L1 could potentially be explored as a biomarker for neuroinflammation.

### CHI3L1 expression in the neuroinflammatory model of SIVE

We have previously reported that CHI3L1 serves as a CSF biomarker for the development of encephalitis after SIV or HIV infection [11]. Our data showed a correlation between the time-course of CHI3L1 concentration and viral load in the CSF. In addition, we found a paradox that CHI3L1 is released from macrophages *in vitro *but *in vivo *CHI3L1 protein was in the soma of astrocytes in the vicinity of microglial nodules. In this study we show that CHI3L1 mRNA is expressed by reactive astrocytes surrounding microglial nodules suggesting that macrophages possibly through released inflammatory mediators, can induce CHI3L1 expression in surrounding astrocytes but not neurons. These findings suggest that CHI3L1 found in the CSF in SIVE/HIVE could be derived from reactive astrocytes. It has been shown by others that differentiated macrophages do express CHI3L1 *in vitro *and *in vivo *in peritumoral macrophages in small cell lung cancer, in atherosclerotic plaques or in an asthma model in mice [12,18,19]. Therefore, it is not clear why CHI3L1 transcription in the brain is less evident in activated tissue macrophages/microglia. We hypothesize that YKL40 transcription by macrophages is inhibited soon after they enter the brain and this may account for the differences seen in other tissue pathologies. We are attempting to further dissect this paradox with in vitro studies.

### CHI3L1 expression in stroke and neurodegenerative diseases

Previously, CHI3L1 expression was shown to be up-regulated in inflammatory conditions, but apart from one report of increased CHI3L1 mRNA in AD brain, there were no data in the literature characterizing the cellular source of CHI3L1 in neurodegenerative diseases. In this study we found high CHI3L1 transcription in two highly inflammatory diseases SIVE and MS. MS is a demyelinating disease associated with waxing and waning inflammation, gliosis and variable axon loss. CSF CHI3L1 levels were significantly elevated in MS patients, however, HIV-infected individual with high viral load showed higher CSF concentration consistent with the observation that neuroinflammation in encephalitis is more robust than in unselected MS. Comparing CSF concentration of CHI3L1 in patients at different stages of MS might be helpful in distinguishing active disease.

CHI3L1 transcript was also found in other neurodegenerative diseases like AD, ALS and Pick's disease as well as stroke, although to a lesser extent than SIVE and MS. In all cases, CHI3L1 was localized to astrocytes from which we would conclude that CHI3L1 expression is a general phenomena of reactive gliosis in response to neuroinflammation. Neuropathological astrocytosis not associated with neuroinflammation (e.g. spontaneous spongiform encephalopathy in rodents) is not associated with YKL40 expression (data not shown). This further emphasizes the use of CHI3L1 as a potential marker for CNS inflammatory conditions with its concentration reflecting a process of reactive gliosis. CSF samples from these diseases show increased CHI3L1 CSF levels compared to healthy young adults. Perhaps not surprisingly, older healthy adults have a very modest but significant elevation in CHI3L1 levels consistent with the hypothesis that low-grade inflammatory processes are induced in the aging brain.

### CHI3L1 expression in schizophrenia

Recent studies showed increased CHI3L1 mRNA in the hippocampus and prefrontal cortex of schizophrenic patients [13,18]. Moreover, two genetic studies claimed that variants in the promoter of CHI3L1 are associated with susceptibility to schizophrenia [19,20]. However, a more recent study failed to confirm a genetic association between CHI3L1 and schizophrenia in Japanese and Chinese populations [21]. Overall, our results showed only occasional CHI3L1 positive cells in schizophrenia. That the younger schizophrenic case (age 32) had almost no CHI3L1 positive astrocytes while the other two cases (ages 52 and 56) had more CHI3L1 positive astrocytes raises the question whether there is a relationship between CHI3L1 expression and schizophrenia or it is just age-related reactive gliosis.

### CHI3L1 expression in acute, subacute and chronic human brain infarction

Comparison of CHI3L1 transcription in acute, subacute and chronic infarcts showed that there is focal and temporal expression of CHI3L1 in astrocytes at the site of injury. Maximal CHI3L1 induction starts at the acute stages (3-5 days; ischemic changes in neurons visible, prominent endothelium and early neutrophilic infiltrate; no reactive astrocytes, no macrophages), continues at the subacute stages (between 5 days to 4 weeks; numerous foamy macrophages, some reactive astrocytes) and then diminished at the chronic stages (more than 4 weeks; few or no macrophages, abundant reactive astrocytes). These differences in YKL-40 transcription in different stages of pathology implies that acute inflammation induces CHI3L1 expression in astrocytes proximal to the lesion and as inflammation resolves CHI3L1 expression is diminished.

## Conclusions

In summary, while the biological functions of CHI3L1 are unclear, its expression is related to inflammation in a variety of diseases, and in this study we show this is true also in the brain. Paradoxically in the brain CHI3L1 synthesis appears to be more astrocytic than macrophage based. The role of CHI3L1 in reactive astrogliosis is still obscure, but from our previous study, it could potentially be involved in growth factor mobilization from the extracellular matrix and attenuation of their biological activities. Alternatively, a recent study by Shao et al showed that CHI3L1 can directly induce cell signaling through syndecan 1 and integrin α_ν_β_3 _and induce focal adhesion kinase phosphorylation [22]. The BRP-39 knockout mice showed reduced inflammation and fibrosis and therefore one might speculate that the expression of CHI3L1 could contribute to the survival of infiltrating inflammatory cells thus prolonging their deleterious effects. This model could be a valuable model to explore the role of CHI3L1 in neuroinflammation.

## Competing interests

The authors declare that they have no competing interests.

## Authors' contributions

DBB conceived the study and participated in the design and coordination of the experiments and drafted the manuscript. GW carried the ISH and immunohistochemistry. AS participated in assessing CSF YKL-40 levels and ISH. RLH assessed the neuropathology of the infarct cases and CAW participated in the design and coordination of the study and helped drafting the manuscript. All authors read and approved the final manuscript.
